# Ochrobactrum anthropi as a Rare Cause of Catheter-Related Bloodstream Infection: A Case Report From Morocco

**DOI:** 10.7759/cureus.98438

**Published:** 2025-12-04

**Authors:** Yousra Boughalem, Youssef Benoumrhar, Youssef El Kamouni, Said Zouhair, Lamiae Arsalane

**Affiliations:** 1 Department of Microbiology, Avicenna Military Hospital, Marrakesh, MAR; 2 Faculty of Medicine and Pharmacy of Marrakech, Cadi Ayyad University, Marrakesh, MAR

**Keywords:** bacteremia, bloodstream infection, catheter-related, hemodialysis, multiple myeloma, ochrobactrum anthropi

## Abstract

*Ochrobactrum anthropi* is a ubiquitous non-fermenting bacillus, rarely implicated in human infections. Reported infections are mostly catheter-related bacteremias, particularly in immunocompromised patients. We present what appears to be the first Moroccan case of catheter-related bloodstream infection associated with *O. anthropi* in a hemodialysis patient with underlying multiple myeloma. The diagnosis was confirmed by identification of the same organism from paired blood cultures, with a differential time to positivity indicating catheter involvement. Treatment included targeted antimicrobial therapy adjusted to renal function, along with catheter removal, which led to clinical improvement of the patient and negative follow-up cultures. This case emphasizes the importance of precise microbiological identification and reliable susceptibility testing to ensure proper management of the patient.

## Introduction

Formerly classified by the Centers for Disease Control and Prevention (CDC) as *Achromobacter* (CDC group Vd) [1,2], *Ochrobactrum anthropi* is now recognized as the type species of the genus *Ochrobactrum* within the Brucellaceae family [2,3]. In 2020, a taxonomic reassignment of *Ochrobactrum* species within the *Brucella* genus was suggested by Hördt et al. [4]. Consequently, *O. anthropi* was renamed *Brucella anthropi* [5]. Currently, both nomenclatures are regarded as valid [6], and our laboratory continues the use of the *Ochrobactrum* nomenclature in routine microbiological practice. 

Despite its wide presence in natural habitats and hospital settings [7], this Gram-negative bacillus is only rarely implicated in human infections [8]. The majority of documented cases are catheter-related bacteremias in immunocompromised patients, particularly those with hematologic malignancies or chronic renal disease necessitating long-term vascular access through catheters [2,7]. *O. anthropi* also expresses an inducible AmpC-like β-lactamase [1], contributing to its challenging antimicrobial management [3] and highlighting the relevance of documenting this case in Morocco, as it improves awareness of this rare pathogen in the region and helps to monitor antimicrobial resistance.

A literature search was conducted using PubMed and Google Scholar search engines, covering the period from August 1980 to September 2025 and using the keywords: (“*Achromobacter* (CDC Vd group)” OR “*Ochrobactrum anthropi*” OR “*Brucella anthropi*”) AND (“catheter-related bloodstream infection” OR “CRBSI” OR “haemodialysis”) AND (“Morocco”). We did not identify any previous publications describing catheter-related bloodstream infections (CRBSI) associated with *O. anthropi* in Moroccan patients undergoing hemodialysis. To our knowledge, we present what appears to be the first Moroccan case of such an infection, occurring in a patient with underlying multiple myeloma. The diagnosis of CRBSI was retained using the differential time to positivity (DTP) criteria, following the guidelines of the Infectious Diseases Society of America (IDSA) [9]. We also describe the microbiological characteristics and antimicrobial susceptibility profile of the isolate. 

## Case presentation

We report the case of an 83-year-old patient admitted to the emergency department with a fever of 38.6 °C that had developed earlier that day. His medical history included type 2 diabetes, well controlled with insulin therapy (glycated hemoglobin 5.2%); end-stage chronic kidney disease, diagnosed 13 years earlier and managed in the nephrology and hemodialysis department with three hemodialysis sessions per week through a tunneled central venous catheter placed three months prior in the right internal jugular vein; and multiple myeloma diagnosed five months earlier, for which he had declined treatment. The patient reported no prior history of catheter-related infection.

The patient’s clinical examination was unremarkable; he was hemodynamically and respiratory stable. Examination of the catheter exit site revealed no erythema, tenderness, or discharge, and palpation down the subcutaneous tunnel was free of induration or pain, indicating no infection of the exit site or tunnel. Chest radiography, transthoracic echocardiography, and abdominal ultrasound were also unremarkable, showing no evidence of deep infectious foci or vegetations. Given this picture of isolated fever in a patient with a tunneled catheter, four sets of blood cultures were collected in parallel, two from the central venous catheter and two others by peripheral venous puncture. Each paired set was collected simultaneously with comparable fill volumes to guarantee accurate assessment of DTP. Additional blood samples were sent to the biochemistry and hematology laboratories for analysis.

Given the clinical presentation characterized by isolated fever in a hemodynamically stable patient and laboratory test results showing neutrophilia and elevated levels of C-reactive protein and procalcitonin, the patient was given symptomatic treatment for his fever and empiric intravenous antibiotic therapy based on amoxicillin-clavulanic acid (1 g/200 mg) at a dosage adapted to his renal function (Table 1). Although immunocompromised, the patient showed no signs of sepsis; examination of the catheter exit site and tunnel was normal, with no history of catheter-related infection and no identifiable source of infection. In this context, amoxicillin-clavulanic acid was regarded as an appropriate empirical choice while waiting for the results of the blood cultures. 

**Table 1:** Laboratory test results on admission. Initial hematological and biochemical parameters obtained upon admission to the hospital included a complete blood count, renal function tests, and infection markers such as C-reactive protein and procalcitonin. eGFR, estimated glomerular filtration rate

Parameters	Results	Reference range
Hemoglobin (g/dL)	10.6	13-18
Mean corpuscular volume (fL)	86.8	78-98
Mean corpuscular hemoglobin concentration (g/dL)	34.3	31-36.5
White blood cells count (cells/µL)	11,390	4,000-11,000
Lymphocytes (cells/µL)	600	1000-4800
Neutrophils (cells/µL)	10,570	1,400-7,700
Eosinophils (cells/µL)	100	20-630
Basophils (cells/µL)	20	0-110
Monocytes (cells/µL)	100	180-1000
Platelet count (G/L)	185	150-450
Blood urea nitrogen (g/L)	1.12	0.15-0.45
Serum creatinine (mg/L)	84.23	6.8-13.6
eGFR (mL/min/1.73 m^2^)	6	80-120
C-reactive protein (mg/L)	37.3	<5
Procalcitonin (ng/mL)	1.2	<0.5

**Figure 1:**
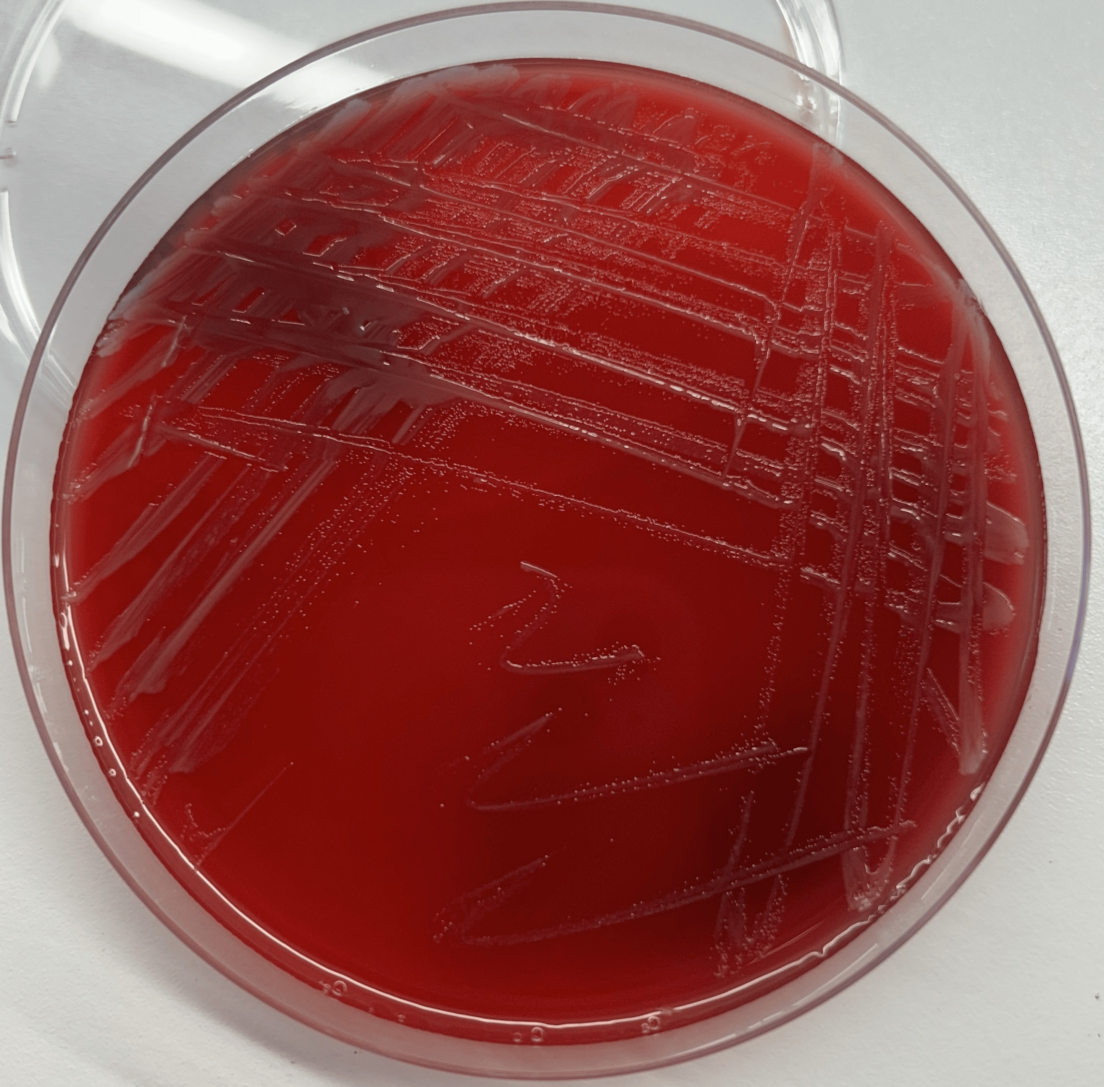
Appearance of Ochrobactrum anthropi colonies on blood agar after incubation for 24 hours at 37 °C under aerobic conditions. The image shows small, mucoid, smooth, slightly yellowish, and non-hemolytic colonies.

**Figure 2:**
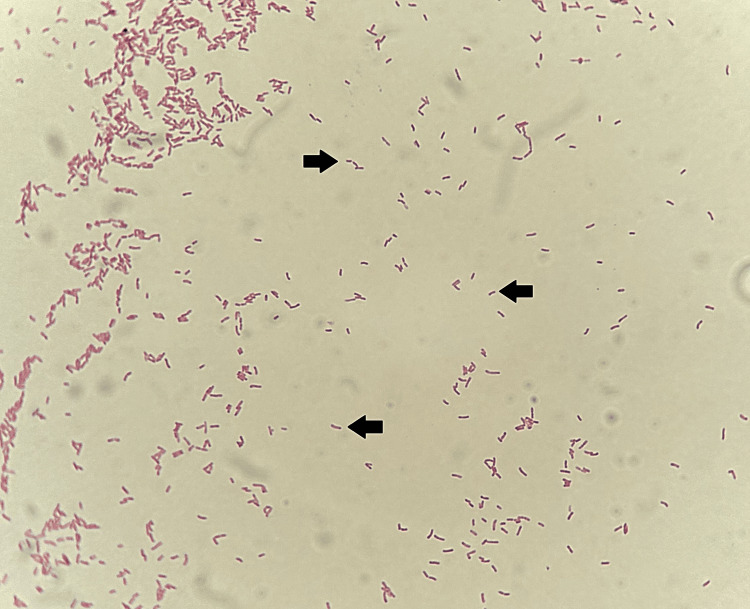
Microscopic appearance of Ochrobactrum anthropi after Gram staining of a slide prepared from a colony (1,000×, oil immersion). The image shows Gram-negative bacilli, some slightly curved (see arrows).

Both aerobic blood culture bottles, drawn from the central venous catheter, turned positive after approximately 48 hours of incubation in the BD BACTEC automated system (Becton, Dickinson and Company, Sparks, MD). Microscopic examination of the blood cultures revealed numerous Gram-negative bacilli. Subculture on blood agar and chocolate agar, incubated for 24 hours in aerobic conditions at 37 °C, revealed small, mucoid, smooth, slightly yellowish, non-hemolytic colonies on blood agar (Figure 1). Microscopic examination of the colonies showed Gram-negative bacilli, some straight and some with a slight curvature (Figure 2), motile in wet preparation. Biochemical characterization showed positive oxidase and catalase tests. Identification was performed using the BD Phoenix M50 system (Becton, Dickinson and Company), which identified *O. anthropi* with a 99% confidence level. This identification was confirmed by an API® 20 NE test strip (bioMérieux, Marcy-l'Étoile, France), giving the numerical profile 1200644 with excellent identification (99.9%).

Furthermore, both peripheral aerobic blood cultures became positive within more than two hours after the corresponding cultures taken from the central venous catheter, thus fulfilling the DTP criteria for CRBSI [9]. Table 2 provides the exact time to positivity (TTP) values and DTP for each paired blood culture set. The isolation of the same species in the peripheral and central blood cultures, confirmed by an identical antimicrobial susceptibility profile, further supported the diagnosis of a CRBSI, in accordance with the IDSA guidelines [9].

**Table 2:** Time to positivity (TTP) and differential time to positivity (DTP) for paired catheter and peripheral blood cultures Exact TTP was reported for each aerobic blood culture, and DTP was calculated for each paired set. A catheter-related bloodstream infection was confirmed when the blood culture collected from the catheter became positive more than two hours earlier than the peripheral culture for both pairs, in accordance with IDSA criteria [9]. IDSA, Infectious Diseases Society of America

Blood culture pair	Source	TTP	DTP	DTP direction
First pair	Catheter	47 hours 53 minutes	2 hours 35 minutes	Catheter 2 hours 35 minutes earlier
	Peripheral	50 hours 28 minutes		
Second pair	Catheter	48 hours 06 minutes	2 hours 43 minutes	Catheter 2 hours 43 minutes earlier
	Peripheral	50 hours 49 minutes		

Antimicrobial susceptibility testing was performed by the disk diffusion method on Mueller-Hinton medium, in accordance with the *Comité de l’Antibiogramme de la Société Française de Microbiologie* (CASFM) and European Committee on Antimicrobial Susceptibility Testing (EUCAST) 2025 guidelines [10]. These standards specify the antibiotics to which the species is known to be naturally resistant or susceptible. Quality control of the method was performed using *Pseudomonas aeruginosa* ATCC 27853 and *Escherichia coli* ATCC 25922 for Trimethoprim-sulfamethoxazole. The tested molecules, the inhibition zone diameters obtained, and their interpretation are presented in Table 3. 

**Table 3:** Antimicrobial susceptibility profile of Ochrobactrum anthropi. Antimicrobial susceptibility testing of the isolated strain. Testing was performed by the disk diffusion method on Mueller-Hinton agar, incubated at 37 °C for 24 hours under aerobic conditions. The table presents inhibition zone diameters (mm) and their interpretation according to CASFM/EUCAST 2025 guidelines [10]. CASFM, Comité de l’Antibiogramme de la Société Française de Microbiologie; EUCAST, European Committee on Antimicrobial Susceptibility Testing

Antibiotic	Inhibition zone diameter (mm)	Interpretation
Amoxicillin-clavulanic acid	0	Resistant
Piperacillin-tazobactam	0	Resistant
Cefoxitin	0	Resistant
Cefixime	0	Resistant
Cefotaxime	0	Resistant
Ceftriaxone	0	Resistant
Ceftazidim	0	Resistant
Cefepime	0	Resistant
Imipenem	32	Susceptible
Meropenem	31	Susceptible
Amikacin	25	Susceptible
Gentamicin	0	Resistant
Ciprofloxacin	32	Susceptible
Tigecycline	30	Susceptible
Trimethoprim–sulfamethoxazole	0	Resistant

Following the antibiogram, a targeted antibiotic therapy was initiated, and the patient was placed on a combination of Imipenem/Cilastatin and Ciprofloxacin for 14 days. Imipenem/Cilastatin was administered at a dose of 500 mg/500 mg every 12 hours, post-dialysis on hemodialysis days and then at 12-hour intervals from the end of the hemodialysis session. Ciprofloxacin was administered intravenously at a dose of 400 mg once a day, post-dialysis on hemodialysis days. Dual therapy was given over monotherapy due to the unpredictable susceptibility profile of *O. anthropi*. Furthermore, the patient's immunocompromised status justified continuing combination therapy, despite timely removal of the catheter, to reduce the risk of therapeutic failure or infection relapse.

**Figure 3:**
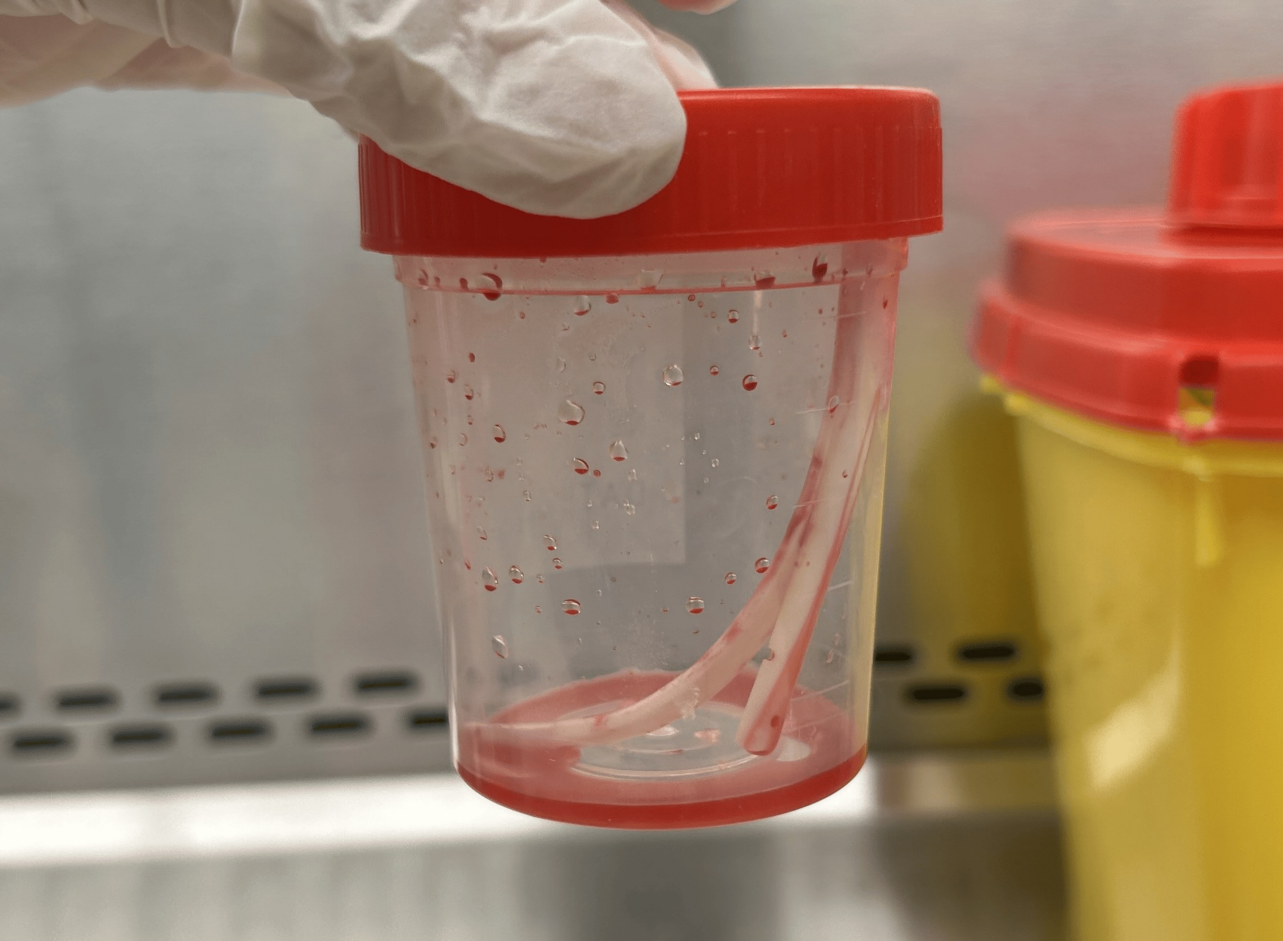
Distal tip of the tunneled catheter sent to the microbiology laboratory of the Avicenne Military Hospital of Marrakesh. Distal tip of the retrieved tunneled central venous catheter, handled in the microbiology laboratory of the Avicenne Military Hospital in Marrakesh. The sample was analyzed using the quantitative culture method of Brun-Buisson [11].

The central venous catheter was removed the next day and replaced with a temporary hemodialysis catheter, then the distal tip of the catheter was sent to the laboratory for bacteriological analysis (Figure 3). The culture was performed on blood agar using the quantitative method of Brun-Buisson [11]. After 24 hours of incubation at 37 °C in aerobic conditions, the culture yielded positive results with 5 x 10^3^ colony-forming units per milliliter (CFU/mL). The identified species and its antibiogram were identical to those of the central and peripheral blood cultures, thus confirming once again the involvement of *O. anthropi* in the CRBSI, according to the same guidelines [6].

After 14 days of targeted antibiotic therapy, the patient's condition improved, with a return to normal body temperature and normalization of infection parameters, including C-reactive protein and procalcitonin. Table 4 reports the C-reactive protein levels at admission, at the start of targeted antibiotic treatment, and on day 14 of treatment.

**Table 4:** C-reactive protein values at admission and during targeted antibiotic therapy. C-reactive protein levels (mg/L) were measured upon admission to the hospital, on the first day of targeted antibiotic treatment, and on the 14th day of treatment. The table illustrates the trend in the inflammatory response during the patient’s hospitalization.

Parameters	On admission	First day of targeted antibiotic therapy	14th day of targeted antibiotic therapy	Reference range
C-reactive protein (mg/L)	37.3	45.5	3	<5

A follow-up blood culture performed at the end of treatment was negative, allowing the reinsertion of a new tunneled catheter to be scheduled. The patient was then discharged and continued his planned hemodialysis sessions at the hospital. Follow-up evaluations at 30 and 90 days showed no recurrence of CRBSI, as the patient remained afebrile and infection markers remained within normal limits.

## Discussion

*O. anthropi* is a Gram-negative, strictly aerobic bacillus, characterized by its non-fermenting metabolism [3,12]. It is mainly found in the natural and hospital environment, particularly in soil and water sources [7]. Although generally recognized as an opportunistic low virulence pathogen, its ability to colonize silicone catheters and form biofilms makes it an increasingly important nosocomial pathogen, particularly in immunocompromised patients [3,7,13].

The first description of *O. anthropi* in humans dates back to 1980, in a pancreatic abscess [14]. Since then, the majority of infections described in the literature were bacteremias, mainly linked to the presence of central venous catheters [3,12]. The microorganism has also been involved in various infections such as pneumonia, endocarditis, peritonitis, endophthalmitis, pelvic abscess, and brain empyema [2]. These infections are generally observed in immunocompromised patients, particularly those with hematologic malignancies, neoplasms, and transplant recipients or those with permanent catheters [7]. Based on our literature search, this observation appears to represent the first documented case of *O. anthropi* CRBSI in Morocco, occurring in a patient with multiple myeloma undergoing hemodialysis via a tunneled central venous catheter. 

CRBSI should always be suspected in hemodialysis patients with fever. The catheter site should be examined, and samples should be taken for bacteriological analysis before starting antibiotics [15]. In our observation, the patient presented with isolated fever in the absence of any apparent source of infection. Furthermore, examination of the catheter showed no signs of inflammation or local infection. The same germ was isolated from both blood culture sites, with a significant DTP between the central venous catheter samples and the peripheral blood samples and precise identification at the species level was achieved using the BD Phoenix M50 system and confirmed with the use of API 20 NE test strips, which allowed us to confirm the diagnosis of CRBSI according to IDSA criteria [9].

*O. anthropi* is typically resistant to most β-lactams, except carbapenems [2]. This resistance is primarily mediated by a chromosomal gene encoding an inducible AmpC-like β-lactamase [1,2]. On the contrary, the species is considered intrinsically susceptible to aminoglycosides, ciprofloxacin, trimethoprim-sulfamethoxazole, and tigecycline [10]. However, susceptibility profiles may be subject to variation from one strain to another [2].

In our case, the isolate was susceptible to amikacin, ciprofloxacin, and tigecycline but showed resistance to gentamicin and trimethoprim-sulfamethoxazole. Although such profiles are not commonly described [2], comparable resistance patterns have been documented in the literature. Zhu et al. analyzed the antimicrobial susceptibility profile of seven *O. anthropi *bloodstream infection isolates and reported susceptibility rates of 85.7% to ciprofloxacin and trimethoprim-sulfamethoxazole, 71.4% to amikacin, and 66.7% to gentamicin [1]. In another study, Thoma et al. determined the antimicrobial susceptibility profile of 63 strains of *O. anthropi* and found that all strains were susceptible to ciprofloxacin, 62 strains were susceptible to trimethoprim-sulfamethoxazole, while only one strain was susceptible to gentamicin [16].

These studies highlight the heterogeneity of resistance profiles within the species, emphasizing the importance of performing antimicrobial susceptibility testing for each isolate. In our case, the results of the testing allowed the clinicians to adjust the patient’s treatment by initiating a targeted antibiotic therapy prompt, which, in association with catheter removal, resulted in clinical improvement of the patient.

## Conclusions

To the best of our knowledge, this appears to be the first reported case of CRBSI associated with *O. anthropi* in a hemodialysis patient in Morocco. The diagnosis was supported by a differential time to positivity showing catheter-drawn blood cultures becoming positive more than two hours earlier than peripheral blood cultures, and was further confirmed by a positive quantitative culture of the catheter distal tip, obtained after rapid source control. Throughout this case, we highlight the importance of precise microbiological identification and reliable susceptibility testing to ensure adequate patient management. Targeted antibiotic treatment was tailored to the patient's renal function and hemodialysis sessions, which resulted in clinical improvement, confirmed by negative follow-up blood cultures. Clinicians need to be aware of this pathogen when dealing with non-fermenting bacteremia in immunocompromised hemodialysis patients, as prompt catheter removal along with targeted antimicrobial therapy is essential for a positive outcome.

## References

[1] Zhu M, Zhao X, Zhu Q, Zhang Z, Dai Y, Chen L, Liang Z (2018). Clinical characteristics of patients with Ochrobactrum anthropi bloodstream infection in a Chinese tertiary-care hospital: a 7-year study. J Infect Public Health.

[2] Ryan MP, Pembroke JT (2020). The genus Ochrobactrum as major opportunistic pathogens. Microorganisms.

[3] Sardana V, Verma SR (2022). Ochrobactrum anthropi: an unusual opportunistic pathogen causing septicemia and pneumonia. IP Int J Med Microbiol Trop Dis.

[4] Hördt A, López MG, Meier-Kolthoff JP (2020). Analysis of 1,000+ type-strain genomes substantially improves taxonomic classification of Alphaproteobacteria. Front Microbiol.

[5] Oren A, Garrity G (2020). List of new names and new combinations previously effectively, but not validly, published. Int J Syst Evol Microbiol.

[6] Moreno E, Middlebrook EA, Altamirano-Silva P (2023). If you're not confused, you're not paying attention: Ochrobactrum is not Brucella. J Clin Microbiol.

[7] Quintela Obregón E, Palomar Fontanet R, Salas C, Rodrigo Calabia E, Arias Rodríguez M (2010). Ochrobactrum anthropi and polymicrobial peritonitis in peritoneal dialysis: a resistance predictor. Nefrologia.

[8] Siti Rohani AH, Tzar MN (2013). Ochrobactrum anthropi catheter-related bloodstream infection: the first case report in Malaysia. Med J Malaysia.

[9] Mermel LA, Farr BM, Sherertz RJ, Raad II, O'Grady N, Harris JS, Craven DE (2001). Guidelines for the management of intravascular catheter-related infections. J Intraven Nurs.

[10] Comité de l’Antibiogramme de la Société Française de Microbiologie (CA-SFM) (2025). Recommandations 2025.

[11] Brun-Buisson C, Abrouk F, Legrand P, Huet Y, Larabi S, Rapin M (1987). Diagnosis of central venous catheter-related sepsis. Critical level of quantitative tip cultures. Arch Intern Med.

[12] Borisov BK, Hitkova HY, Linkova SP (2021). Tunnelled hemodialysis catheter-related bloodstream infection with Ochrobactrum anthropi: a report of the first two cases from Bulgaria and a brief overview. Folia Med (Plovdiv).

[13] Alnor D, Frimodt-Møller N, Espersen F, Frederiksen W (1994). Infections with the unusual human pathogens Agrobacterium species and Ochrobactrum anthropi. Clin Infect Dis.

[14] Appelbaum PC, Campbell DB (1980). Pancreatic abscess associated with Achromobacter group Vd biovar 1. J Clin Microbiol.

[15] Beaudreuil S, Hebibi H, Charpentier B, Durrbachr A (2008). Les infections graves chez les patients en dialyse péritonéale et en hémodialyse chronique conventionnelle: péritonites et infections de la voie d’abord vasculaire. Réanimation.

[16] Thoma B, Straube E, Scholz HC (2009). Identification and antimicrobial susceptibilities of Ochrobactrum spp. Int J Med Microbiol.

